# Arrhythmic risk during pregnancy and postpartum in patients with long QT syndrome

**DOI:** 10.1007/s00399-021-00757-4

**Published:** 2021-03-29

**Authors:** Babken Asatryan, Marina Rieder, Alessandro Castiglione, Katja E. Odening

**Affiliations:** 1 Translational Cardiology, Department of Cardiology, Inselspital, Bern University Hospital, Freiburgstrasse 8, 3010 Bern, Switzerland; 2 Institute of Physiology, University of Bern, Buehlplatz 5, 3012 Bern, Switzerland

**Keywords:** Cardiac arrhythmia, Genetics, Sex-differences, Postpartum, Sudden cardiac death, Herzrhythmusstörungen, Genetik, Geschlechtsunterschiede, Postpartale Phase, Plötzlicher Herztod

## Abstract

Congenital long QT syndrome (LQTS) is a genetic disorder characterized by a prolonged QT interval in the surface electrocardiogram (ECG) that predisposes affected individuals to arrhythmic syncope, ventricular torsades-de-pointes, and sudden cardiac death at a young age. Investigations of large patient cohorts revealed sex-related differences in the LQTS phenotype. Adult women with LQTS are at higher risk for cardiac arrhythmias than are adult men with LQTS. Sex hormones are thought to play the primary role for these gender differences. Clinical experience and translational studies indicated that females with LQTS have a lower risk for cardiac arrhythmias during pregnancy and elevated risk in the postpartum period due to contrasting effects of estradiol and progesterone, as well as postpartum hormones on the action potential and arrhythmia substrate. However, this pro- or anti-arrhythmic potential of hormones varies depending on the underlying genotype, partly since sex hormones have distinct effects on different (affected) cardiac ion channels. Thus, a comprehensive evaluation of women with LQTS prior to and during pregnancy, during labor, and in the postpartum period with consideration of the patient’s disease- and gene-specific risk factors is essential to providing precision management in this patient group. This review discusses the current understanding of hormonal influences in LQTS and provides practical guidance for the optimal management of LQTS patients during pregnancy, delivery, and the postpartum period.

## Abstract (de)

Das kongenitale Long-QT-Syndrom (LQTS) ist eine genetische Erkrankung, die durch ein verlängertes QT-Intervall im Oberflächenelektrokardiogramm (EKG) charakterisiert ist und Betroffene zu arrhythmogenen Synkopen, ventrikulären Torsades-de-pointes-Tachykardien und plötzlichem Herztod im jungen Alter prädisponiert. Untersuchungen an großen Patientenkohorten zeigten geschlechtsspezifische Unterschiede im LQTS-Phänotyp: Erwachsene Frauen mit LQTS haben ein höheres Risiko für Herzrhythmusstörungen als gleichaltrige Männer. Für diese geschlechtsspezifischen Unterschiede im LQTS-Phänotyp werden primär Geschlechtshormone verantwortlich gemacht. Klinische Erfahrungen und translationale Studien deuten zudem darauf hin, dass Frauen mit LQTS in der Schwangerschaft ein geringeres Risiko für Herzrhythmusstörungen haben, während das Risiko in der Zeit nach der Geburt erhöht ist. Diese Veränderungen im arrhythmogenen Risiko werden gegensätzlichen Effekten von Östradiol und Progesteron sowie Effekten postpartaler Hormone auf Aktionspotenzial und arrhythmogenes Substrat zugeschrieben. Das pro- bzw. antiarrhythmische Potenzial der Hormone variiert abhängig vom zugrunde liegenden Genotyp, teilweise weil Geschlechtshormone unterschiedliche Effekte auf die verschiedenen (betroffenen) kardialen Ionenkanäle haben. Daher ist eine genaue Einschätzung vor und während der Schwangerschaft, während der Geburt und in der postpartalen Phase mit Bewertung der patienten-, krankheits- und gen-spezifischen Risikofaktoren essenziell, um ein präzises Management von Frauen mit LQTS zu gewährleisten. Die vorliegende Übersicht stellt das gegenwärtige Wissen über hormonelle Einflüsse beim LQTS dar und bietet praktische Empfehlungen zum optimalen Management von Patientinnen mit LQTS während der Schwangerschaft, bei der Geburt und in der postpartalen Phase.

## Introduction

Congenital long QT syndrome (LQTS) is a genetic disorder characterized by a prolonged QT interval in the surface electrocardiogram (ECG) that predisposes affected individuals to arrhythmic syncope, ventricular torsades-de-pointes (TdP), and sudden cardiac death (SCD) at a young age [31]. Pathogenic variants in three genes are known to account for about 90% of all LQTS cases. *KCNQ1* (LQT1) and *KCNH2* (LQT2) encode the pore-forming subunits of voltage-gated repolarizing K^+^ channels conducting I_Ks_ and I_Kr,_ respectively, whereas *SCN5A* (LQT3) encodes the pore-forming subunit, Na_V_1.5, of the cardiac sodium channel conducting I_Na_ and the pathological late I_Na,L_ in LQTS [31].

## Changes in QT duration and arrhythmic risk during pregnancy and postpartum in women with LQTS

In patients with LQTS, pronounced sex-related differences can be observed in the heart-rate corrected QT (QTc) and arrhythmic risk, strongly suggesting an important role for female and male sex hormones. Adult women with congenital or acquired LQTS have longer QTc intervals and a higher risk for arrhythmic events such as TdP and SCD than men [31, 39]. In women with congenital LQTS, the risk for arrhythmias is reduced during pregnancy, while it is particularly high during the postpartum period [36]. Pronounced genotype-related differences were observed in this risk of postpartum-related arrhythmias with a particularly high risk in patients with LQT2 [36], in which the arrhythmic risk was found to be up to four times higher than before pregnancy [10, 36]. Notably, up to 10% of all women with LQTS experience their first cardiac event during the postpartum period [28]. Finally, in women with drug-induced LQTS, QT-prolongation and the arrhythmic risk were more pronounced in the follicular than the luteal phase of the menstrual cycle [30], a hormonal change that still needs to be confirmed for women with congenital LQTS. These data imply that the different sex hormone concentrations (and progesterone/estradiol ratios) at different phases of the menstrual cycle, pregnancy, and postpartum affect the QT duration and the arrhythmic risk in LQTS.

**Fig. 1:**
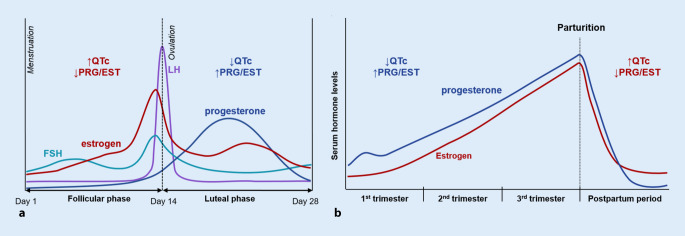
Variation in female hormone levels during the menstrual cycle (**a**) and during pregnancy and the postpartum period (**b**). *QTc* corrected QT, *PRG* progesterone, *EST* estrogen, *FSH* follicle stimulating hormone

The female menstrual cycle has follicular and luteal phases. The follicular phase is characterized by low levels of progesterone and a continuous rise in estradiol with a pre-ovulatory peak. The transition from the follicular to the luteal phase is characterized by a peak in luteinizing hormone (LH), which induces ovulation. In the luteal phase, progesterone and estrogen levels show a peak about 1 week after ovulation (however, the estrogen peak is smaller than that during the follicular phase), followed by a rapid decrease in the premenstrual days (Fig. 1; [37]).

Pregnancy is characterized by an increase in estrogen and progesterone with a rapid decline of both hormone levels in the early postpartum period [41]. In breastfeeding women, the normal ovarian cycle is disrupted by the release of gonadotropin-releasing hormone and LH, resulting in reduced follicular estradiol production with consequently reduced estradiol levels [41].

Relevant changes in the QT interval are observed during the menstrual cycle, with shorter QT observed in the luteal than in the follicular phase—in both congenital as well as drug-induced LQTS, attributed mainly to the increased progesterone levels during the luteal phase (Fig. 1; [21, 30]). The opposing effects of estradiol and progesterone on QT could also be observed in women with postmenopausal hormone treatment: unopposed estrogen supplementation in menopausal women prolonged QT, and the effect was reversed by the addition of progesterone [9]. In line with this, a case report focusing on QT duration in a woman with LQTS during pregnancy and postpartum showed a temporal shortening/normalization of the QTc duration owing to increased progesterone levels during pregnancy and while using a progesterone-releasing intra-uterine device [25].

These clinical observations were complemented by experimental studies in transgenic LQT2 rabbit models to further elucidate the underlying mechanisms by which hormones may impact cardiac repolarization (as described in detail in the following section) [23]. In LQT2 rabbits, estradiol consistently prolonged while progesterone consistently shortened QT duration. Also, estradiol was shown to act as a pro-arrhythmic modulator in LQTS by increasing the incidence of lethal ventricular arrhythmia due to changes in the electrical substrate and due to increased susceptibility to pro-arrhythmic sympathetic triggers; in contrast, progesterone had an anti-arrhythmic, protective disease-modulating effect in LQT2 rabbits [23]. These anti-arrhythmic progesterone effects may thus contribute to the reduced arrhythmic risk during pregnancy (in which high progesterone levels are observed) [30, 36].

The increased arrhythmic risk during the postpartum period may be partly due to the marked decrease in anti-arrhythmic progesterone concentration. Recent experimental evidence in transgenic LQT2 rabbits, however, suggests that the postpartum-related hormones oxytocin and prolactin may act as additional endogenous pro-arrhythmic disease modifiers by prolonging cardiac repolarization [2].

## (Hormonal) Mechanisms underlying changes in QT duration and arrhythmic risk during pregnancy and postpartum in women with LQTS

**Fig. 2:**
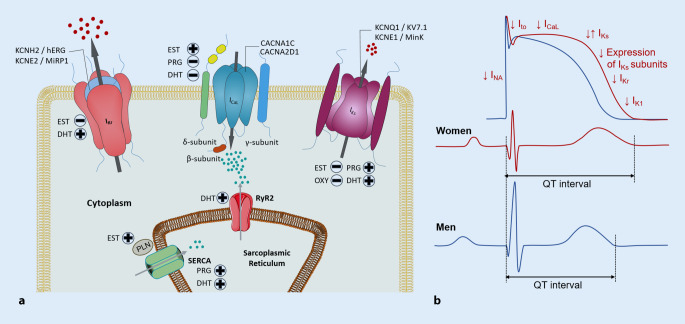
Molecular mechanisms underlying the hormonal effects on cardiac ion channels (**a**) and their effect on different phases of the action potential (**b**). *CACNA1C/CACNA2D1* α- and β‑subunits of L‑type calcium channel, *DHT* dihydrotestosterone, *EST* estradiol, *ICa,L* L-type Ca2+ current, *IK1* inward rectifier K+ current, *IKr* rapid delayed rectifier K+ current, *IKs* slow delayed rectifier K+ current, *KCNE1/MinK* β-subunit to KCNQ1/Kv7.1 to form slow delayed rectifier K+ current, *KCNE2/MiRP1* β-subunit to HERG/KCNH2 to form slow delayed rectifier K+ current, *PLN* phospholamban, *PRG* progesterone, *RyR2* ryanodine receptor, *SERCA* sarcoplasmic reticulum ATPase, + increase, − decrease

Different mechanisms have been proposed to explain how sex hormones and changes in their concentration may impact cardiac repolarizing ion currents, calcium handling properties, and resulting changes in cardiac repolarization/QT duration and arrhythmic risk (Fig. 2). Due to ethical considerations, most of these studies have been conducted on different animal models for LQTS. In addition to mice and guinea pigs, rabbit models have played a pivotal role as they show close similarity to humans in repolarizing ion currents, in the sex-related differences in cardiac repolarization, and in LQTS-related arrhythmias [3, 14, 22].

In addition to changing sex hormone levels, other internal or external factors may alter cardiac electrophysiology during pregnancy and postpartum, such as alterations in adrenergic activity and a disrupted sleep pattern, particularly during postpartum, and thus contribute to changes in arrhythmic risk during pregnancy and postpartum [28, 34]. As these mechanisms are not yet well investigated, the focus here is on summarizing direct hormone effects on cardiac electrophysiology.

## Estradiol

Estradiol has been demonstrated to increase the arrhythmic risk in LQTS by prolonging QT, especially at slow heart rates [23]. Experimental data suggest that estradiol interacts with several ion currents: it inhibits I_Kr_ through direct interaction with the human ether-à-go-go-related gene (hERG) channel (via the aromatic group of phenylalanine in position 656 of the channel protein) [12]. Moreover, estradiol reduces the transcription of *KCNE2*, the β‑subunits to the hERG/I_Kr_ channel via a genomic estrogen-receptor mediated mechanism [11].

In addition, estradiol affects Ca^2+^ handling. It upregulates ryanodine receptor 2 (RyR2) expression, which leads to higher Ca^2+^-mediated triggered activity [15]. Estradiol affects the amplitude of I_Ca,L_ currents by increasing its channel expression, thereby enhancing the propensity to develop early afterdepolarizations [23, 40].

## Progesterone

In contrast to estradiol, progesterone exerts beneficial QT-shortening effects, thus decreasing the arrhythmic risk in LQTS [23, 35]. Through a non-genomic acute pathway, progesterone enhances I_Ks_ and inhibits I_Ca,L_ in a concentration-dependent fashion [5, 6]. Progesterone increases the intracellular Ca^2+^ reuptake in the sarcoplasmic reticulum by increasing the expression and activity of the sarcoplasmic calcium ATPase (SERCA), which may relevantly contribute to the antiarrhythmic effect exerted by progesterone [20].

## Testosterone

The blood concentration of the male sex hormone testosterone increases by 60% from the first to the third pregnancy trimester and may therefore also contribute to QT changes during pregnancy [32]. Testosterone acutely shortens ventricular action potentials by increasing repolarizing potassium currents I_Kr_, I_Ks_, and I_K1_ via a non-genomic pathway [7, 13]. Additionally, testosterone chronically increases I_Ks_ currents through upregulation of Kv7.1 (*KCNQ1*) mRNA expression [16].

Contrasting acute and chronic testosterone effects on Ca^2+^ currents are observed: while testosterone acutely inhibits I_Ca,L_ [5], it chronically increases L‑type calcium channel (Ca_V_1.2) expression, thus exerting differential effects on cardiac repolarization [19]. Also, testosterone enhances SERCA function, leading to faster Ca^2+^ reuptake and hence an anti-arrhythmic shortening of Ca^2+^ transient duration, but it also enhances the activity of RyR2 [38].

## Oxytocin

Oxytocin is released in a pulsatile manner during labor and breast feeding. Data on its role in cardiac electrophysiology stem from studies in rabbit models. In the first studies, any direct oxytocin effects on hERG, Nav1.5, or Ca_V_1.2 were excluded [27]. More recently, in transgenic LQT2 rabbits, an oxytocin-induced prolongation of QTc/action potential duration (APD) due to direct acute oxytocin-induced inhibition of I_Ks_ was identified [2]. This blockade of I_Ks_ does not only explain the QT/APD prolonging effect of oxytocin, but may also account for the genotype differences in postpartum-related arrhythmias with particularly high risk in LQT2, in which, due to the loss of I_Kr_, I_Ks_ represents the main repolarizing current. Also, these data suggest that peaking levels in blood oxytocin may lead to increased arrhythmia susceptibility in LQTS.

## Prolactin

Data concerning the potential impact of prolactin on cardiac electrophysiology is limited. Similar to oxytocin, QT-prolonging effects could be observed in transgenic LQT2 rabbits in vivo and ex vivo due to I_Ks_-blocking properties of prolactin [2].

## Recommendations for LQTS patients during pregnancy, delivery, and postpartum

As female LQTS patients are particularly susceptible to life-threatening arrhythmias in phases associated with changing hormone levels, close clinical monitoring of these women during pregnancy, labor, and the postpartum period is critical. In general, a multidisciplinary team approach is necessary to evaluate and carefully manage pregnant women with LQTS. Currently, management is based on the assessment of individual as well as gene- and disease-specific risk factors, avoidance of arrhythmia triggers, and—very importantly—uninterrupted β‑blocker therapy during pregnancy and the postpartum period [1], which significantly reduces the risk of arrhythmias and SCD [36].

The choice of β‑blockers is less established due to the limited evidence available today; while more data exists for *fetal safety* of metoprolol and propranolol [29], propranolol or nadolol seem to be the *most effective* in reducing the arrhythmic risk [8]. For these reasons, propranolol is the most widely used for LQTS and often the preferred β‑blocker in pregnancy and the postpartum period at 2–3 mg/kg/day [33]. Generally, β‑blockers are associated with slightly lower fetal birth weights, but are otherwise well tolerated during pregnancy and the postpartum period [17].

Mothers with LQTS have an eight-fold increased risk for stillbirth and a two-fold higher risk for miscarriages as compared to the general population [4]. More stringent follow-up of LQTS women during pregnancy might therefore be necessary, particularly when severe and sustained sinus bradycardia (defined as fetal heart rate ≤ 3rd percentile for gestational age) is observed [18]. In one study, TdP and II° atrioventricular (AV) block occurred in around 24% of all fetal LQTS and were highly specific for fetal LQTS [18]. Interestingly, fetal LQT1 seemed to show a mild phenotype with sinus bradycardia, whereas individuals with LQT3 or genotype-negative LQTS tended to develop TdP and/or II° AV block [18].

In patients considered to be at high risk for malignant ventricular arrhythmias, the availability of a cardiologist and maternal cardiac telemetry during labor is advisable. Unassisted vaginal delivery can be safely performed in women with LQTS, particularly in those with an implantable cardioverter defibrillator (ICD). However, decisions regarding the delivery plan in LQTS women should be individualized to the maternal risk profile as, in those with a history of arrhythmia, the risk of life-threatening arrhythmias during labor is likely elevated. Anesthetic planning should include reviewing the list of medications to avoid potentially QT-prolonging drugs (for the full list see https://crediblemeds.org) [24].

Although arrhythmias in LQT1 and LQT2 are commonly provoked by high sympathetic tone [26], arrhythmic events appear to be rare during labor. The highest heart rates during labor are observed in the active pushing phase. Notably, heart rate increases more in those receiving intravenous oxytocin, which additionally also prolongs cardiac repolarization and can predispose LQTS patients to TdP arrhythmia. Therefore, oxytocin should preferably be avoided in LQTS patients.

## Concluding remarks and perspectives

In women with LQTS, pregnancy, delivery, and the postpartum period are critical phases, with a variation of arrhythmia risk associated with changing hormonal levels. While most LQTS patients have a reduced risk for arrhythmias and SCD during pregnancy, the risk for arrhythmias drastically increases in the postpartum period, in particular in women with LQT2, necessitating close patient monitoring and management. Female LQTS patients should be informed that uninterrupted β‑blocker therapy is the key to eventless pregnancy, delivery, and postpartum. A multidisciplinary approach to women with LQTS involving a cardiologist/cardiac electrophysiologist, geneticist, as well as an obstetrics/gynecology specialist ensures comprehensive risk assessment and optimal patient care. Although on the one hand, female sex hormones pose a challenge in the management of LQTS women, their differential effects on cardiac ion channels offer the potential for developing sex hormone-based anti-arrhythmic therapies. While much remains to be understood about the complex interaction of sex hormones and cardiac ion currents/action potential, and their extra-cardiac effects, translational studies serve as a model framework for exploring these novel therapeutic avenues.

## Declarations

For this review article no studies with human participants or animals were performed by any of the authors. All studies mentioned were performed in accordance with the ethical standards indicated in each case.

## References

[1] Asatryan B, Yee L, Ben-Haim Y (2021). Sex-related differences in cardiac Channelopathies: implications for clinical practice. Circulation.

[2] Bodi I, Sorge J, Castiglione A (2019). Postpartum hormones oxytocin and prolactin cause pro-arrhythmic prolongation of cardiac repolarization in long QT syndrome type 2. Europace.

[3] Brunner M, Peng X, Liu GX (2008). Mechanisms of cardiac arrhythmias and sudden death in transgenic rabbits with long QT syndrome. J Clin Invest.

[4] Cuneo BF, Kaizer AM, Clur SA (2020). Mothers with long QT syndrome are at increased risk for fetal death: findings from a multicenter international study. Am J Obstet Gynecol.

[5] Er F, Michels G, Brandt MC (2007). Impact of testosterone on cardiac L-type calcium channels and Ca2+ sparks: acute actions antagonize chronic effects. Cell Calcium.

[6] Furukawa T, Kurokawa J (2008). Non-genomic regulation of cardiac ion channels by sex hormones. Cardiovasc Hematol Disord Drug Targets.

[7] Furukawa T, Kurokawa J (2007). Regulation of cardiac ion channels via non-genomic action of sex steroid hormones: implication for the gender difference in cardiac arrhythmias. Pharmacol Ther.

[8] Ishibashi K, Aiba T, Kamiya C (2017). Arrhythmia risk and beta-blocker therapy in pregnant women with long QT syndrome. Heart.

[9] Kadish AH, Greenland P, Limacher MC (2004). Estrogen and progestin use and the QT interval in postmenopausal women. Ann Noninvasive Electrocardiol.

[10] Khositseth A, Tester DJ, Will ML (2004). Identification of a common genetic substrate underlying postpartum cardiac events in congenital long QT syndrome. Heart Rhythm.

[11] Kundu P, Ciobotaru A, Foroughi S (2008). Hormonal regulation of cardiac KCNE2 gene expression. Mol Cell Endocrinol.

[12] Kurokawa J, Kodama M, Clancy CE (2016). Sex hormonal regulation of cardiac ion channels in drug-induced QT syndromes. Pharmacol Ther.

[13] Liu XK, Katchman A, Whitfield BH (2003). In vivo androgen treatment shortens the QT interval and increases the densities of inward and delayed rectifier potassium currents in orchiectomized male rabbits. Cardiovasc Res.

[14] Liu XK, Wang W, Ebert SN (1999). Female gender is a risk factor for torsades de pointes in an in vitro animal model. J Cardiovasc Pharmacol.

[15] Long V, Fiset C (2019). Contribution of estrogen to the pregnancy-induced increase in cardiac automaticity. J Mol Cell Cardiol.

[16] Masuda K, Takanari H, Morishima M (2018). Testosterone-mediated upregulation of delayed rectifier potassium channel in cardiomyocytes causes abbreviation of QT intervals in rats. J Physiol Sci.

[17] Meidahl Petersen K, Jimenez-Solem E, Andersen JT (2012). β-Blocker treatment during pregnancy and adverse pregnancy outcomes: a nationwide population-based cohort study. Bmj Open.

[18] Mitchell JL, Cuneo BF, Etheridge SP (2012). Fetal heart rate predictors of long QT syndrome. Circulation.

[19] Montano LM, Calixto E, Figueroa A (2008). Relaxation of androgens on rat thoracic aorta: testosterone concentration dependent agonist/antagonist L-type Ca2+ channel activity, and 5beta-dihydrotestosterone restricted to L-type Ca2+ channel blockade. Endocrinology.

[20] Moshal KS, Zhang Z, Roder K (2014). Progesterone modulates SERCA2a expression and function in rabbit cardiomyocytes. American journal of physiology. Am J Physiol.

[21] Nakagawa M, Ooie T, Takahashi N (2006). Influence of menstrual cycle on QT interval dynamics. Pacing Clin Electrophysiol.

[22] Odening KE, Castiglione A (2020). Acquired long QT syndrome and sex hormones. Sex and cardiac electrophysiology.

[23] Odening KE, Choi BR, Liu GX (2012). Estradiol promotes sudden cardiac death in transgenic long QT type 2 rabbits while progesterone is protective. Heart Rhythm.

[24] Odening KE, Hyder O, Chaves L (2008). Pharmacogenomics of anesthetic drugs in transgenic LQT1 and LQT2 rabbits reveal genotype-specific differential effects on cardiac repolarization. Am J Physiol Heart Circ Physiol.

[25] Odening KE, Koren G, Kirk M (2016). Normalization of QT interval duration in a long QT syndrome patient during pregnancy and the postpartum period due to sex hormone effects on cardiac repolarization. Heart Rhythm Case Rep.

[26] Priori SG, Wilde AA, Horie M (2013). HRS/EHRA/APHRS expert consensus statement on the diagnosis and management of patients with inherited primary arrhythmia syndromes: document endorsed by HRS, EHRA, and APHRS in May 2013 and by ACCF, AHA, PACES, and AEPC in June 2013. Heart Rhythm.

[27] Qu Y, Fang M, Gao B (2015). Oxytocin does not directly alter cardiac repolarization in rabbit or human cardiac myocytes. Pharmacol Res Perspect.

[28] Rashba EJ, Zareba W, Moss AJ (1998). Influence of pregnancy on the risk for cardiac events in patients with hereditary long QT syndrome. LQTS Investigators. Circulation.

[29] Regitz-Zagrosek V, Roos-Hesselink JW, Bauersachs J (2018). 2018 ESC Guidelines for the management of cardiovascular diseases during pregnancy. Eur Heart J.

[30] Rodriguez I, Kilborn MJ, Liu XK (2001). Drug-induced QT prolongation in women during the menstrual cycle. JAMA.

[31] Sauer AJ, Moss AJ, Mcnitt S (2007). Long QT syndrome in adults. J Am Coll Cardiol.

[32] Schock H, Zeleniuch-Jacquotte A, Lundin E (2016). Hormone concentrations throughout uncomplicated pregnancies: a longitudinal study. BMC Pregnancy Childbirth.

[33] Schwartz PJ (2013). Practical issues in the management of the long QT syndrome: focus on diagnosis and therapy. Swiss Med Wkly.

[34] Schwartz PJ, Priori SG, Spazzolini C (2001). Genotype-phenotype correlation in the long-QT syndrome: gene-specific triggers for life-threatening arrhythmias. Circulation.

[35] Sedlak T, Shufelt C, Iribarren C (2012). Sex hormones and the QT interval: a review. J Womens Health.

[36] Seth R, Moss AJ, Mcnitt S (2007). Long QT syndrome and pregnancy. J Am Coll Cardiol.

[37] Sherman BM, Korenman SG (1975). Hormonal characteristics of the human menstrual cycle throughout reproductive life. J Clin Invest.

[38] Tsang S, Wong SS, Wu S (2009). Testosterone-augmented contractile responses to alpha1- and beta1-adrenoceptor stimulation are associated with increased activities of RyR, SERCA, and NCX in the heart. Am J Physiol Cell Physiol.

[39] Vink AS, Clur SB, Wilde Aa M (2018). Effect of age and gender on the QTc-interval in healthy individuals and patients with long-QT syndrome. Trends Cardiovasc Med.

[40] Yang X, Chen G, Papp R (2012). Oestrogen upregulates L-type Ca(2)(+) channels via oestrogen-receptor- by a regional genomic mechanism in female rabbit hearts. J Physiol.

[41] Zinaman MJ, Cartledge T, Tomai T (1995). Pulsatile GnRH stimulates normal cyclic ovarian function in amenorrheic lactating postpartum women. J Clin Endocrinol Metab.

